# Acquired Uterine Arteriovenous Malformation Following Dilatation and Curettage Treated with Bilateral Uterine Artery Embolization: A Case Report

**DOI:** 10.7759/cureus.4250

**Published:** 2019-03-13

**Authors:** Salman Khan, Shakir Saud, Imran Khan, Basit Achakzai

**Affiliations:** 1 Internal Medicine, Guthrie Clinic/Robert Packer Hospital, Sayre, USA; 2 Family Medicine, Rutgers New Jersey Medical School, Newark, USA; 3 Internal Medicine, North Shore University Hospital, Hempstead, USA; 4 Interventional Radiology, Guthrie Clinic/Robert Packer Hospital, Sayre, USA

**Keywords:** uterine arteriovenous malformation, uterine artery embolization, dilatation and curettage, gelfoam pledgets

## Abstract

Uterine arteriovenous malformations (AVMs) are a rare, potentially life-threatening cause of abnormal uterine bleeding that can be acquired following uterine instrumentation. We herein report a case of acquired uterine AVM following dilatation and curettage (D&C) that was successfully treated with bilateral embolization using Gelfoam (Pfizer, New York, USA) pledgets.

## Introduction

Arteriovenous malformation (AVM) is an abnormal connection between the arteries and veins without an intervening capillary bed. When present within the uterus, it will present as a recurrent, often profuse vaginal bleeding that can result in life-threatening hemorrhage without intervention. AVMs can be congenital or acquired [1]. Acquired AVMs are often the consequence of previous uterine trauma, such as curettage procedures, cesarean section, or pelvic surgery [2]. Treatment depends on the symptoms, age, desire for future fertility, localization, and size of the lesion. Embolization of the uterine artery is an efficacious and effective method of treating AVM, particularly in patients of reproductive age.

## Case presentation

A 30-year-old G1P0010 presented to the emergency department with a six-day history of abnormal uterine bleeding. Three months prior, she underwent a therapeutic abortion followed by dilatation and curettage (D&C) for retained products of conception. Since then, she was noted to have new-onset menorrhagia, which on the day of presentation became persistent and was associated with severe pain, weakness, and dizziness. She was found to be hypotensive and tachycardic on presentation with marked tenderness in the suprapubic area with an otherwise normal physical exam. Laboratory studies revealed hemoglobin of 9.2 g/dL and hematocrit of 27.5%, and negative beta-human chorionic gonadotropin (beta-hCG).

**Figure 1:**
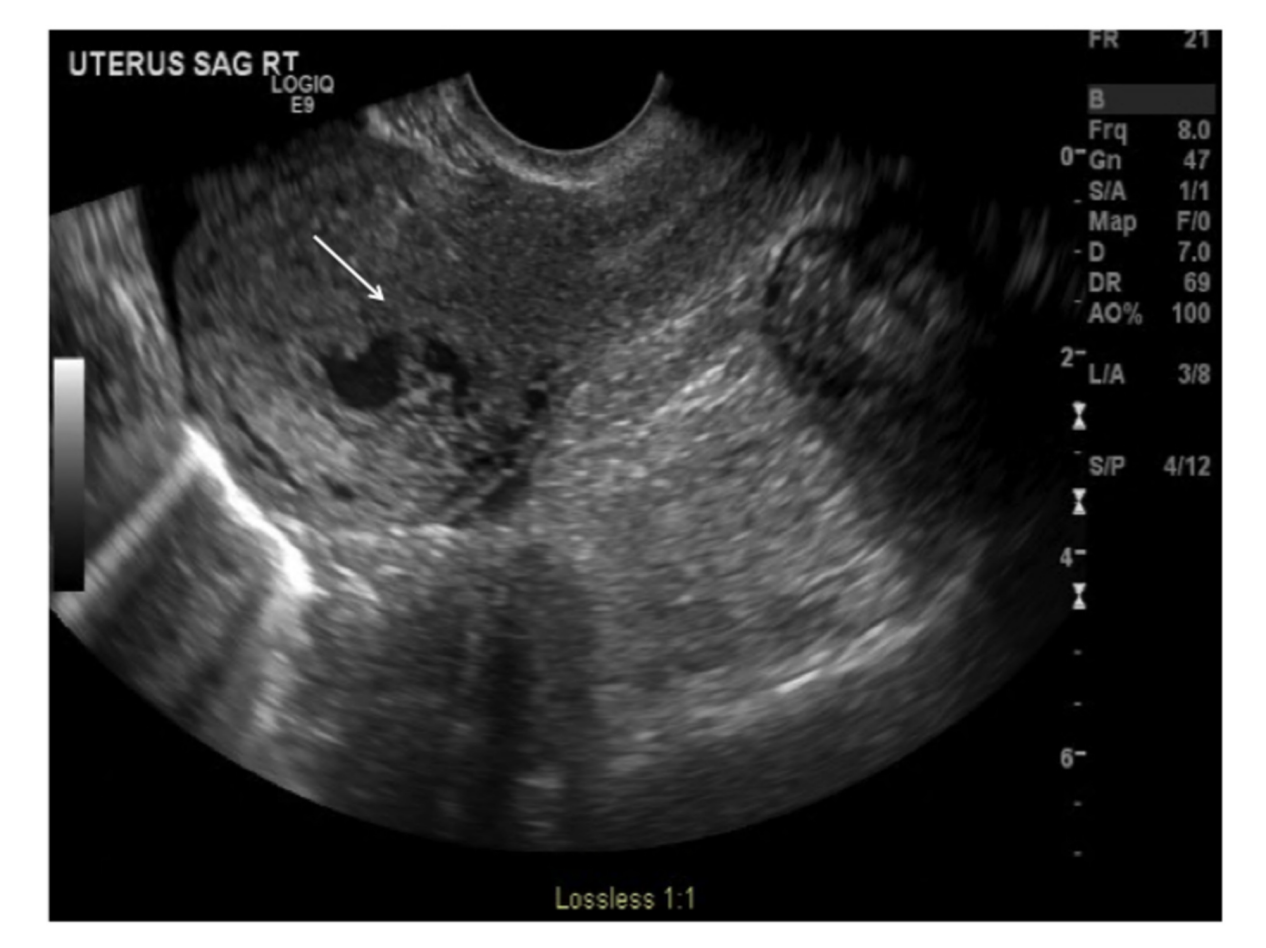
Transvaginal ultrasound of the uterus showing a hypoechoic lesion in the body of the uterus

**Figure 2:**
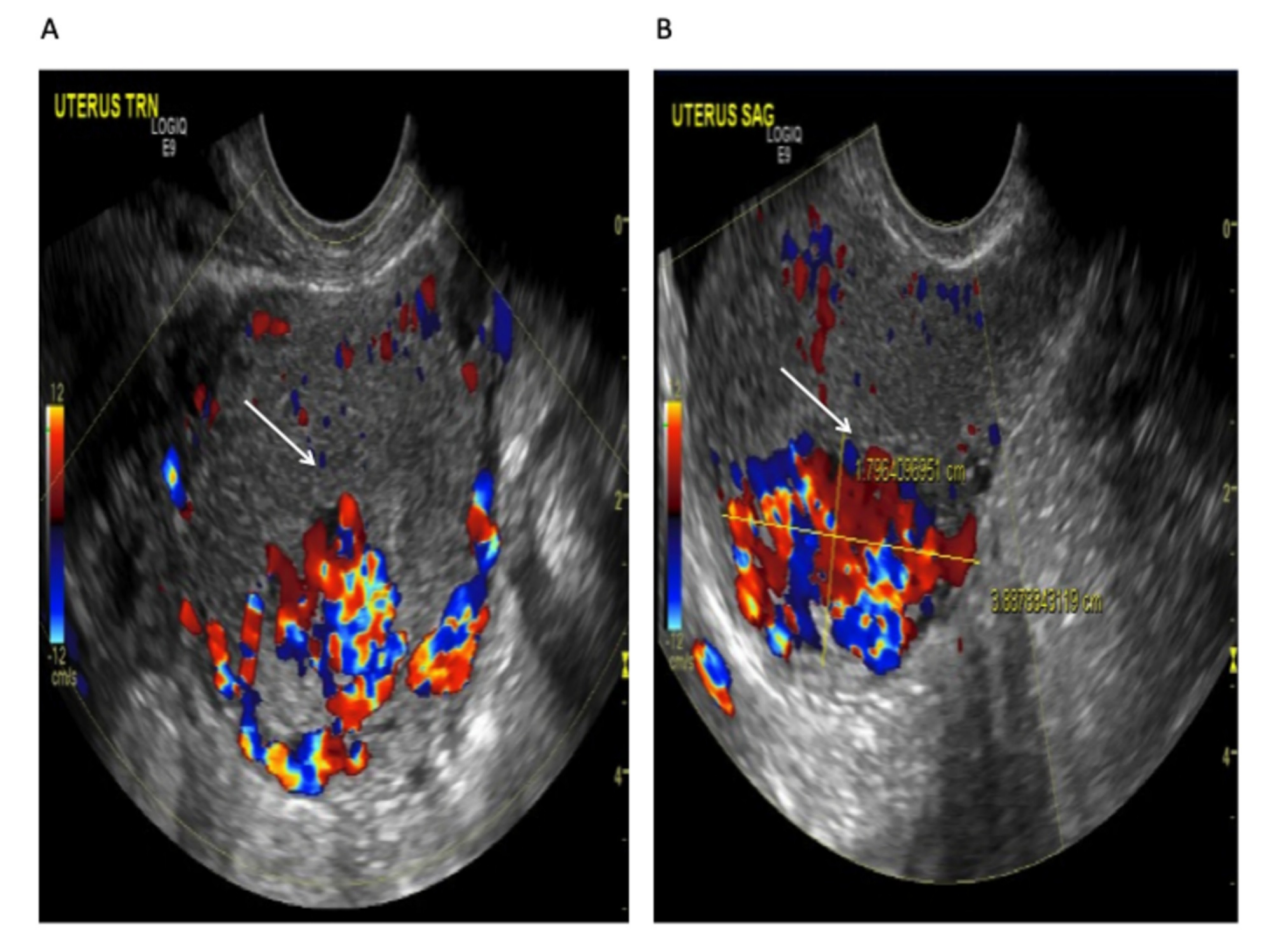
Transvaginal ultrasound of the uterus with Doppler revealing a hypervascular lesion: (A) transverse view (B) sagittal view

**Figure 3:**
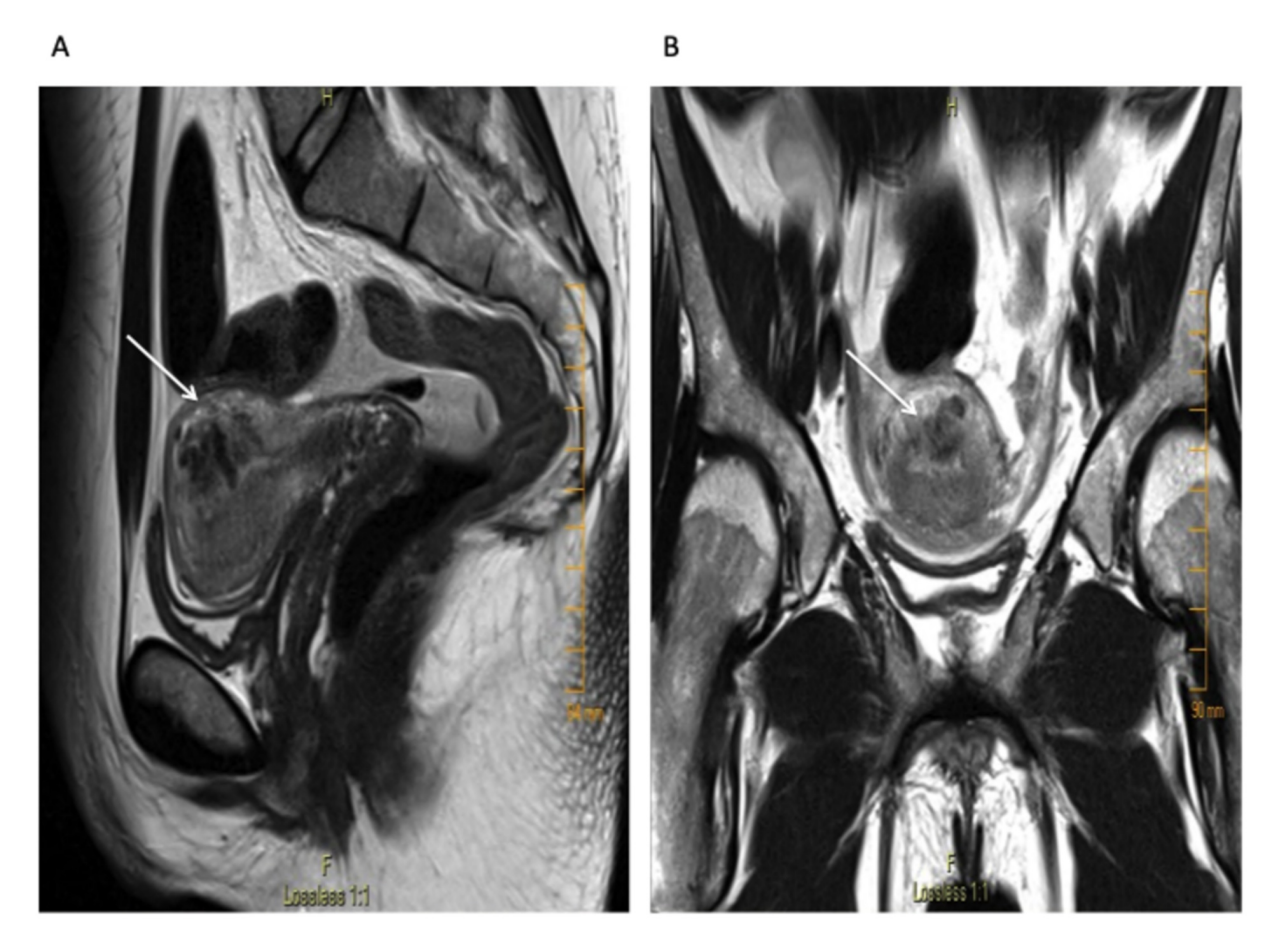
T2-weighted pelvic magnetic resonance imaging (MRI) revealing a hypointense T2 lesion within the posterosuperior wall of the myometrium: (A) sagittal view (B) axial view

Transvaginal ultrasound revealed a 3.9 cm x 2.7 cm x. 1.8 cm sized anechogenic cystic space in the posterior wall of the fundus as seen in Figure 1, and with Doppler application, demonstrated marked vascularity (Figure 2). For further evaluation, pelvic magnetic resonance imaging (MRI) was performed, which revealed a contrast-opacified structure within the wall of the myometrium. Focal serpiginous flow voids were also noted within the posterosuperior myometrium with extension to the myometrial canal (Figure 3).

**Figure 4:**
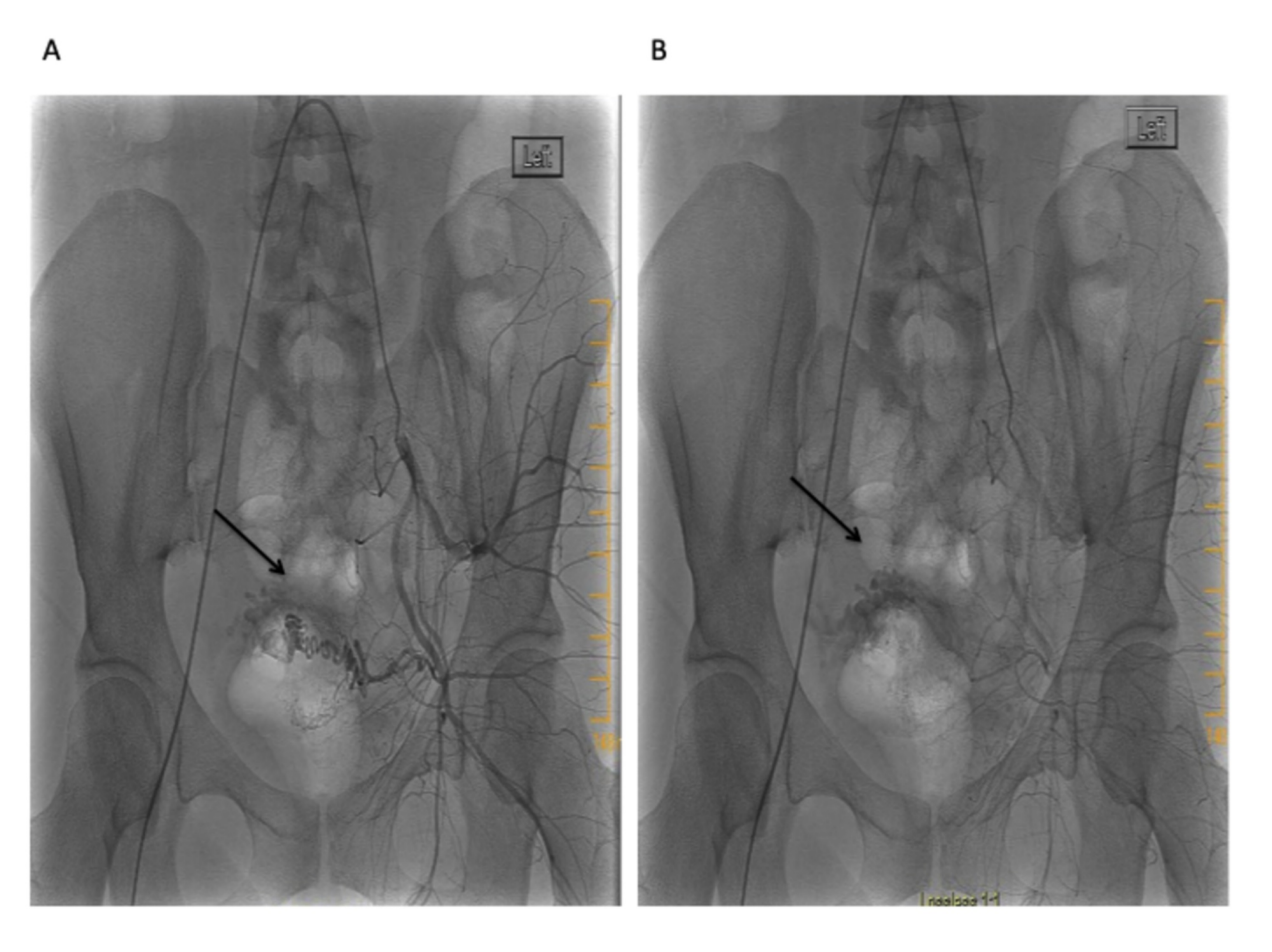
Left internal iliac artery angiogram revealing a hypervascular lesion within the uterine artery distribution with early venous filling during the late arterial phase: (A) early arterial phase (B) late arterial phase

Taken together, a presumptive diagnosis of the uterine AVM was made and the patient was referred to interventional radiology. The uterine angiogram confirmed the presence of an AVM within the posterosuperior wall (Figure 4). There were multiple feeding arteries mainly from the left with smaller arteries from the right. Embolization of both uterine arteries was performed with Gelfoam (Pfizer, New York, USA) pledgets to near stasis. The post-embolization arteriogram showed complete embolization of the AVM with slow flow of contrast in both uterine arteries. No immediate complications were encountered. The patient's vaginal bleeding resolved and she was discharged three days later. No recurrence of abnormal uterine bleeding was reported at either the three-week or six-month follow-up visit.

## Discussion

Uterine arteriovenous malformations are a rare cause of abnormal uterine bleeding. With the increased utilization of surgical gynecology, the prevalence of acquired uterine AVM will likely increase. Thus, uterine AVM is an important differential that should be considered in any patient who presents with abnormal uterine bleeding. The first case of uterine AVM was described in 1926 and to date, there are fewer than 100 reported cases in the literature [3-4]. In the present case, a D&C was likely the inciting event that led to the formation of an acquired uterine AVM. Acquired AVMs can also be associated with cesarean section, pelvic surgery, infection, gestational trophoblastic disease and some gynecologic malignancies [5].

In the past, AVMs were difficult to diagnose and was often found upon tissue examination post-hysterectomy. The availability of color Doppler ultrasound has transformed the diagnosis of AVM [6], which is now a non-invasive modality that can aid in detecting this rare condition [7]. A confirmatory test is often followed as retained products of conception, hemangioma, gestational trophoblastic disease, and complex ovarian cyst can give a hypervascular appearance with a turbulent flow on Doppler [5], thus digital subtraction angiography remains the gold standard [8].

Traditionally, hysterectomy was the preferred modality in the treatment of uterine AVMs; however, uterine artery embolization (UAE) is increasingly becoming one of the preferred treatment methods partly due to the effectiveness, minimally invasive nature, and possibility of preserving uterine function to allow future childbearing [9]. Studies have shown bilateral UAE to be 90% percent effective; with technical failures being attributed to procedure complications, incomplete embolization and uterine artery rupture [10]. Various embolic materials have been used, including polyvinyl alcohol, histoacryl glue, stainless steel coils, detachable balloons, and hemostatic gelatine [5]; however, no study has compared the efficacy between the different methods. In the present case, Gelfoam pledgets were used to successfully embolize the AVM without complication or the need for re-embolization.

This case highlights both the diagnostic advances in identifying AVMs and demonstrating the effectiveness of using Gelfoam pledgets as a single embolic agent for urgent bilateral UAE in the treatment of AVM.

## Conclusions

Bilateral UAE with Gelfoam pledgets is a safe, effective, and minimally invasive method to treat uterine AVMs with long-term efficacy, which can provide the preservation of fertility.
